# Aspartame and Phenylketonuria: an analysis of the daily phenylalanine intake of aspartame-containing drugs marketed in France

**DOI:** 10.1186/s13023-023-02770-x

**Published:** 2023-06-08

**Authors:** Victor Maler, Violette Goetz, Marine Tardieu, Abderrahmane El Khalil, Jean Meidi Alili, Philippe Meunier, François Maillot, François Labarthe

**Affiliations:** 1 Reference Center for Inborn Errors of Metabolism ToTeM, CHRU de Tours, Hôpital Clocheville, 49 Bd Béranger, 37000 Tours, France; 2 Pharmacie à Usage Intérieur, Hôpital Clocheville, 49 Bd Béranger, 37000 Tours, France; 3 Filière G2m, Hôpital Necker-Enfants Malades, APHP, 149 Rue de Sèvres, 75015 Paris, France; 4 INSERM U1253, iBrain, Université François Rabelais de Tours, 10 Boulevard Tonnellé, 37000 Tours, France; 5 INSERM U1069, Nutrition, Croissance et Cancer, Faculté de Médecine, Université François Rabelais de Tours, 10 Boulevard Tonnellé, 37000 Tours, France

**Keywords:** Phenylketonuria, Phenylalanine, Aspartame, Sugar tax, Restricted diet, Metabolic control

## Abstract

**Background:**

Phenylketonuria (PKU) is a rare genetic metabolic disorder in which especially high phenylalanine (Phe) concentrations cause brain dysfunction. If untreated, this brain dysfunction results in severe microcephaly, intellectual disability, and behavioral problems. Dietary restriction of Phe is the mainstay of PKU treatment, with long-term successful outcomes. Aspartame, an artificial sweetener sometimes added into medications, is metabolized in the gut into Phe. Then, patients suffering from PKU on a Phe-restricted diet should avoid consumption of aspartame. The aim of our study was to evaluate the number of drugs containing aspartame and/or Phe as an excipient, and to quantify their corresponding Phe intake.

**Methods:**

The list of drugs marketed in France containing aspartame and/or Phe was established using a national medication database called “Theriaque”. For each drug, the corresponding daily Phe intake was calculated according to age and weight and was distributed into 3 categories: high (> 40 mg/d), medium (10 to 40 mg/d) and low (< 10 mg/d) Phe intake.

**Results:**

The number of drugs containing Phe or its precursor aspartame remained very limited (*n* = 401). Among the aspartame containing drugs, Phe intakes were significant (medium or high) for only half of them whereas there were negligible for the others. Furthermore, these medications with a significant Phe intake were limited to few pharmaceutical classes (mainly antiinfectives agents, analgesics, and drugs for nervous system), and within these classes the drugs were limited to a small number of molecules, including principally amoxicillin, amoxicillin + clavulanic acid and paracetamol/ acetaminophen.

**Discussion:**

In situations requiring the use of these molecules, we propose as an alternative, the use of an aspartame-free form of these molecules or a form with a low Phe intake. If it is not possible, we propose as second-line the use of another antibiotics or analgesics. Finally, we have to remember the benefits-risk balance to use medications containing significant Phe intake in PKU patients. Indeed, it may be better to use a Phe containing medication in the absence of an aspartame-free form of this drug rather than to leave a person with PKU without treatment.

**Supplementary Information:**

The online version contains supplementary material available at 10.1186/s13023-023-02770-x.

## Background

Phenylketonuria (PKU, MIM#261,600, ORPHAcode 716) is a rare genetic metabolic disease caused by the deficiency of the enzyme phenylalanine hydroxylase (EC 1.14.16.1) that converts phenylalanine (Phe) to tyrosine. This pathology induces a blockage of the metabolism pathway of Phe, causing its accumulation, particularly in the blood and in the brain [1, 2]. Untreated, PKU is characterized by irreversible intellectual disability, microcephaly, developmental problems and psychiatric symptoms. As high blood Phe concentrations are strongly related to neurocognitive outcome, existing treatments aim at decreasing blood Phe concentrations. The classical treatment of PKU relies mainly on a low protein diet aimed to restrict the daily Phe-intake [3]. Most patients on diet treatment tolerate less than 500 mg/d of Phe, with a minimum about 200 mg/d [4]. Besides this controlled diet, other therapeutic options also exist. Some patients are responsive to and are treated with BH4, acting as a pharmaceutical chaperone, which allows a higher natural protein intake [1]. However, only a subset of patients with PKU respond to this treatment. More recently, an enzyme replacement therapy using pegylated Phe ammonia lyase has been approved by FDA and EMA [1]. Adult PKU patients treated with this therapy can be free of dietary control with an improved metabolic control. Other non-nutritional treatment approaches, including gene therapy and therapeutic liver repopulation, have not progressed currently beyond animal models [1, 2]. The monitoring of PKU patients is based on the dosage of blood Phe concentration, whose target levels varying with age and correlated with neurocognitive outcome.

Aspartame is an artificial sweetener that can be added to soft drinks, foods and also to some medications in order to improve their palatability without sugar intake [5, 6]. It is metabolized in the gastrointestinal tract into Phe, aspartic acid and methanol. Aspartame is therefore a source of Phe that should be excluded from food and drug for PKU patients. In France, the label on aspartame-containing drugs includes a warning, mentioning that the use of these drugs is contraindicated or must be made with cautions in people suffering from PKU, but without specifying the importance of the Phe intake they provide [7, 8]. A list of aspartame containing drugs is available for patients in some countries, including France, but is also a source of stress and anxiety for patients and/or their parents [9, 10]. Recent European PKU guidelines recommend that for immediate and short-term treatment of infections, if only aspartame containing medicines are available, it may be better to use these until aspartame-free medication is sourced rather than leave a patient with PKU without treatment as blood Phe levels will rise with infection [1, 2]. However, for chronic use of medications, it is better to find alternative aspartame free medications.

The aim of our study was to evaluate the number of drugs containing aspartame and/or Phe as an excipient, and to quantify their corresponding Phe intake (when used at a standard posology) in order to assess if this Phe intake is relevant or not considering metabolic control. We also proposed alternative medications for commonly used drugs with a significant Phe intake.

## Methods

### Data selection

The list of drugs marketed in France containing aspartame and/or Phe was established by using the French database “Theriaque” (https://www.theriaque.org) with the keywords “aspartame”, “phenylalanine” and “phenylcetonurie”. Thereafter, we excluded drugs with an exceptional use, such as vaccines (unique dose) or chemotherapies to treat cancer or equivalent (for which the benefit-risk balance is in strong favor of the use of these drugs), as well as a drug that was no longer marketed. Then, we removed all the drugs for which the quantity of aspartame or Phe was not specified in the composition.

### Calculation of the daily Phe intake

The determination of the daily Phe intake of each drug was carried out according to the information on their composition in excipient. The amount of Phe used for the analysis was the amount specified in the composition of the drug (in mg of Phe per g of the active substance for Phe-containing drug). For aspartame containing drugs, we used the stoichiometric equation for aspartame metabolism, considering that the complete metabolism of 1 g of aspartame results into an equimolar amount of 0.56 g of Phe [3, 5]. Then, we calculated the corresponding amount of Phe for each drug used at its conventional posology (in mg/kg/d for children and/or in mg/d for adults). When different doses exist according to their medical indications, the maximal posology was selected. Therefore, the daily Phe-intake of each drug (in mg/d) was calculated for an adult patient, and according to weight for children using a large range of theorical weights (including 5, 10, 20, 30, 40, 60, 80 and 100 kg). For simplification, only results for 10, 20 and 30 kg of weight are presented, because for a weight of 5 kg the Phe intake was very low, and that, beyond 30 kg, the Phe intake values reached those of adults.

### Constitution of Phe intake categories

To assess the magnitude of the daily Phe intake of each drug, three categories have been arbitrarily established. A “low Phe intake” was defined as a daily Phe intake < 10 mg/d, which was considered as negligible amounts, equivalent to 5% or lower of the minimum daily Phe intake corresponding to the strictest diet (dietary restriction about 200 mg/d) [4]. The “medium Phe intake” and the “high Phe intake” were defined as between 10 and 40 mg/d and > 40 mg/d, respectively. For each selected medication, the results of its daily Phe intake is presented for a child weighting 10, 20 or 30 kg and for an adult, with the distribution of each result in the 3 categories “high”, “medium” or “low” Phe intake. To describe the distribution of the different drugs in each categories, we use the pharmaceutical Anatomical Therapeutic Chemical (ATC) classification available on the WHO website (https://www.whocc.no/atc_ddd_index/).

## Results

### Quantitative analysis

**Fig. 1:**
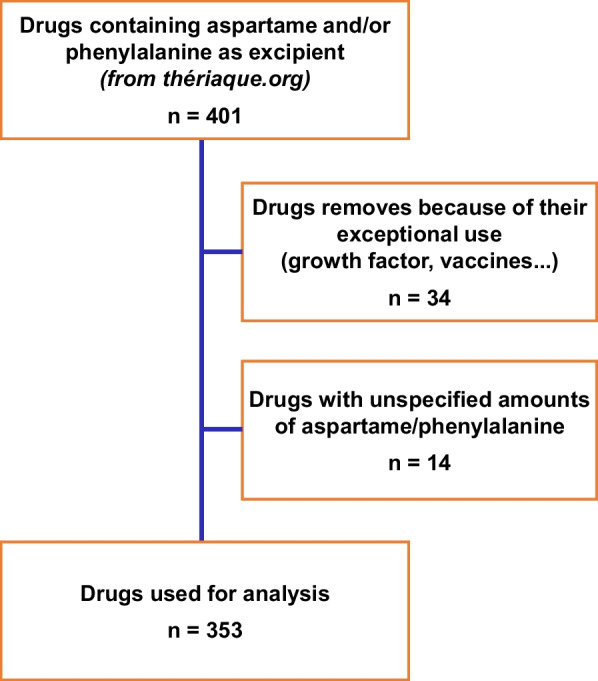
Flowchart of the selection of drugs containing Phe and/or aspartame

We identified 401 drugs containing aspartame and/or Phe as excipient (see flowchart, Fig. 1). Thirty-four drugs were excluded because of their exceptional use (21 hematopoiesis stimulating factors and 10 vaccines, both containing Phe, one chemotherapy and one immunosuppressive treatment containing aspartame, and a drug that was no longer marketed), and 14 additional drugs because we were unable to find the amount of aspartame or Phe in their composition. The final analysis was done on a total of 353 drugs marketed in France and containing aspartame. Among these drugs, 134 were indicated in children and adults, 13 only in children and 206 only in adults (Table 1). The drugs were sorted according to their calculated daily Phe-intake at conventional posology. The majority of drugs had a low Phe-intake index (< 10 mg/d), especially in youngest children, representing about two third of the drugs. However, the number of drugs containing aspartame with low daily Phe-intakes in children declined with increasing weight, from 63% for a weight of 10 kg to 44% for a weight of 30 kg. On the other hand, the number of drugs providing a high daily Phe-intake (> 40 mg/d) remained relatively limited, with only 9 (6%) drugs in 10 kg children versus 50 (33%) drugs in children weighting 30 kg. The number of aspartame-containing drugs that brought a medium daily Phe-intake was more fluctuating, representing from 23 to 41% of the selected drugs in children. The distribution in adults remained relatively similar with 176 (52%) drugs with a low Phe-intake, 71 (21%) with a medium and 90 (27%) with a high Phe-intake.

**Table 1:** Distribution of daily Phe intakes of aspartame-containing drugs

	Body weight (for children)			Adults
	10 kg	20 kg	30 kg	
Low daily Phe intake (< 10 mg/d)	94 (63%)	68 (45%)	66 (44%)	176 (52%)
Medium daily Phe intake (10 – 40 mg/d)	47 (31%)	61 (41%)	34 (23%)	71 (21%)
High daily Phe intake (> 40 mg/d)	9 (6%)	21 (14%)	50 (33%)	90 (27%)
Total	150	150	150	337

Results are presented in number of drugs (%). The daily Phe intake of each drug was calculated for a use at conventional posology according to age and weight. We considered that the complete metabolism of 1 g of aspartame results into an equimolar amount of 0.56 g of Phe

### Qualitative analysis

**Fig. 2:**
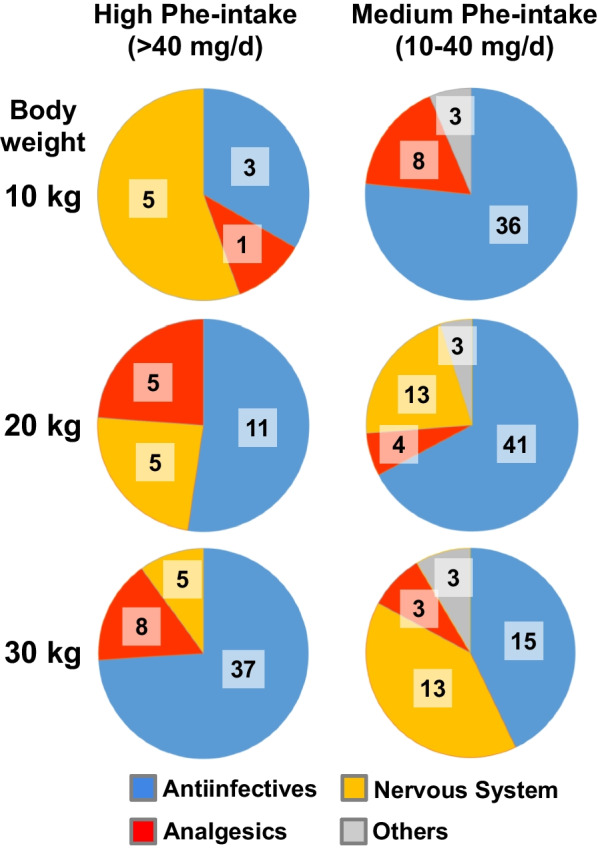
Distribution by pharmacologic classes of aspartame containing drugs with a high or medium Phe intake in children. The daily Phe intake of each drug was calculated for a use at conventional posology according to age and weight. We considered that the complete metabolism of 1 g of aspartame results into an equimolar amount of 0.56 g of Phe. High and medium Phe intakes were defined as > 40 mg/d and between 10 and 40 mg/d, respectively. The different drugs were distributed according to the ATC classification available on the WHO website (see “Methods” section for details). The number of drugs for each condition is noted on the graph

In a second part of the analyses, we described the distribution of the drugs according to their pharmacological classes. For children (Fig. 2), antiinfectives, which was exclusively represented by the antibiotic amoxicillin ± clavulanic acid for a weight of 30 kg or lower, was the most important class of drugs with a high or medium Phe-intake whatever the children weight. The other classes of drugs were mostly analgesics (exclusively acetaminophen/paracetamol) and drugs targeting central nervous system (risperidone and lamotrigine). Note that for children weighing 10 kg, neurological drugs accounted for 56% (*n* = 5) of the high Phe-intake drugs. However, this corresponds to 5 different forms of risperidone, a medication without indication before the age of 5y, and then with an improbable use in a child weighting 10 kg or lower. Indeed, antibiotics should be considered as the most important drug family with a significant Phe-intake also in youngest patients. Three other drugs also contributed to a medium Phe-intake, including a medication for diarrhea (diosmectite), another for constipation (a mix of lactulose, paraffin and vaseline), and one form of prednisolone tablets. To summarize in children, drugs with a high intake of Phe were limited to principally amoxicillin ± clavulanic acid, acetaminophen/paracetamol, risperidone and lamotrigine.

**Fig. 3:**
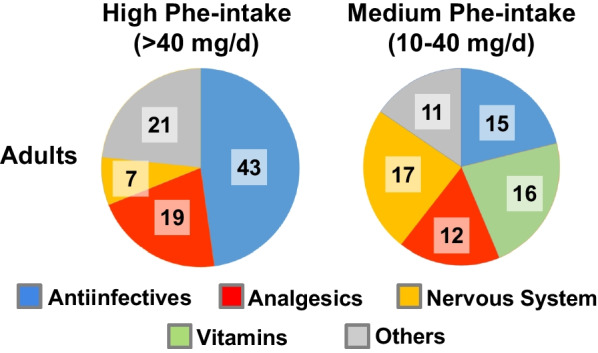
Distribution by pharmacologic classes of aspartame containing drugs with a high or medium Phe intake in adults. The calculated daily Phe intake of each drug and the distribution of drugs were performed as previously described. The number of drugs for each condition is noted on the graph

The distribution of drugs with high Phe-intake was more various in adults (Fig. 3). Antibiotics remained the main pharmacological class with 43 (48%) medications with a high Phe-intake and 15 (21%) with a medium Phe-intake. It included mainly amoxicillin ± clavulanic acid (which represented 52 of the 58 antiinfectives with high and medium Phe-intake), but also 3 macrolide or related drugs, 2 forms of 2nd generation cephalosporins, and one topical antiseptic for pharyngitis. Analgesic drugs (acetaminophen/ paracetamol and Non-Steroidal Anti-Inflammatory Drugs, NSAIDs) represented the second group of drugs with 19 (21%) and 12 (17%) medications with a high or medium Phe-intake, respectively. Neurological drugs (principally risperidone), with 7 (8%) and 17 (24%) medications with a high or medium Phe-intake, and vitamins (*n* = 16, 23% of the drugs with medium Phe-intake) were also present. Finally, other drugs (principally for gastroenterological and respiratory symptoms) represented 23% (*n* = 21) and 15% (*n* = 11) of the medications with a high or medium Phe-intake, respectively.

### Main medications containing significant amounts of aspartame

**Fig. 4:**
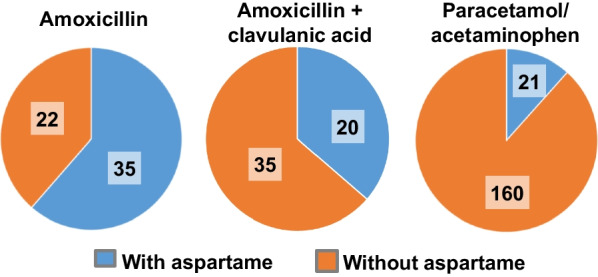
Distribution of marketed forms of amoxicillin, amoxicillin + clavulanic acid and paracetamol/acetaminophen according to their aspartame contain. The number of drugs for each condition is noted on the graph

The main drugs with a common use associated with a significant Phe-intake were amoxicillin, the association amoxicillin + clavulanic acid and paracetamol/acetaminophen. For each one, we reported the proportion of marketed forms with and without aspartame (Fig. 4). About two third of the commercialized forms of amoxicillin contained aspartame (35 from 57), whereas it was only 36% of the available forms of amoxicillin + clavulanic acid (20 from 55) and 12% of the commercialized forms of paracetamol/acetaminophen (21 from 181). In the aim to propose alternative solutions, we identified several forms of each drug without aspartame and with a galenic form adapted to age and weight (see Additional file 1).

## Discussion

PKU patients who are treated with dietary Phe-restriction should avoid consumption of aspartame. Aspartame is a Phe-containing synthetic sweetener used in many products, including many soft drinks and dietary products and also several medications. Aspartame is metabolized in the gastrointestinal tract into three components: Phe, aspartic acid and methanol. In the present study, we firstly identified all marketed drugs available in France and containing aspartame and/or Phe as excipient. These medications represented only a small proportion of all drugs marketed in France, with 401 drugs containing aspartame and/or Phe for more than 13 000 different drug presentations sold in pharmacies in 2021 [11], representing about 3% of all medications. After exclusion of exceptional drugs (mainly hematopoiesis stimulating factors and vaccines) and of 14 additional drugs with unknown Phe contain, it remained 353 medications as potential source of Phe, all of them containing aspartame. This list of medications is available for patients and their parents/carers, and also for prescribers, with the instruction that if suitable aspartame-free medication cannot be sourced, it is better to use aspartame-containing medications (notably antibiotics) rather than leave a patient without treatment.

The originality of our work was to estimate for each drug from this list, the corresponding daily quantity of Phe when the drug is used at regular posology (according to weight and age). Using this methodology, the proportion of the medications that contain aspartame with a significant daily Phe-intake (arbitrary defined > 10 mg/d of Phe) represented only a limited portion of the drugs from this list (about 37% of the aspartame-containing drugs for child weighting 10 kg, 55% for 20 kg, 56% for 30 kg and 48% for adults). In children, the number of medications containing significantly Phe increased with weight, considering that dose was in mg/kg/d whereas the target of Phe intake was in mg/d, irrespective of weight. For a weight of 40 kg or over, posology joined the adult ‘one.

Furthermore, medications with a significant Phe intake came from only a limited number of pharmacological classes, namely antiinfectives, analgesics and drugs targeting central nervous system drugs. More in details, it concerns only few drugs commonly used in children, including amoxicillin (with or without clavulanic acid) and acetaminophen/paracetamol that represent all the significant Phe-containing antiinfectives (*n* = 52) and analgesics (*n* = 11) drugs respectively, and risperidone which corresponds to the majority of the drugs targeting the central nervous system (*n* = 15 from 18, 83%). The distribution of significant Phe-containing medications was more various in adults, also including besides the previous medications a few numbers of other antibiotics, aspirin and other NSAIDs, and several forms of vitamins. However, some pathologic conditions may require chronic medications in adult PKU patients, in contrast to children for whom most of the treatments are for acute conditions. More in details, adult patients with PKU can be individualized in two different groups. The first one includes early treated patients who we may except to enjoy reasonable health, with perhaps some complications of PKU subject to treatment exposure [1, 2, 12]. The second group corresponds to late treated PKU patients who might be having a restricted Phe intake, but still require drugs targeting central nervous system, such as antiepileptic drugs [1, 2, 13]. In such situations, a chronic intake of Phe-containing drugs may be challenging and may expose some susceptible individuals to adverse reactions caused by disturbances in their metabolic control. It is important for the carers and patients that all measures are taken to optimise metabolic control, even in patients who have established brain damage. This study offers the opportunity to simplify the list of medications to avoid for PKU patients (limiting to only few drugs). Accidental aspartame consumption due to medications is not exceptional in PKU patients, because aspartame is commonly used as a sugar replacement in medicines. In a recent online survey, 23% of PKU patients or their parent/carers said that they had been prescribed medicines containing aspartame by their doctors, and that medicines were not checked when prescribed [9]. Respondents felt there was little awareness or concern about the presence of aspartame in medications amongst medical professionals when they prescribed medication for PKU. Generally, reminders to check prescriptions for aspartame came from patients/parents’ instruction rather than the GP or pharmacist. A simplified list of “significant Phe-intake” drugs could be useful to prevent accidental aspartame consumption due to medications.

The second aim of the present work was to propose alternative aspartame-free treatments to limit Phe-intake due to medication. Firstly, we reported that the majority of the marketed forms of amoxicillin contained aspartame (*n* = 35, i.e. 61%). The majority of powders for drinkable suspensions have quantities of aspartame corresponding to a medium to high Phe-intake. They are mostly for pediatric use, and the addition of aspartame as a sweetener is aimed to improve palatability of the medication without increasing sugar consumption, notably to reduce the risk of dental caries [6]. However, we identified several galenic forms adapted to age and weight and aspartame-free. Three tablet forms of amoxicillin also contained aspartame, but in negligible amounts. Thus, several approaches may be proposed to PKU patients. It is possible to establish a list of marketed forms of amoxicillin without aspartame (*n* = 22/57), or with a negligible daily quantity of Phe-intake according to weight. Then, the use of adult-forms like tablets may be suggested but will require performing a dilution to obtain the appropriate dosage. An example of proposition is presented in the Additional file 1. Another solution is to recommend the use of an alternative antibiotic, as it has been proposed for amoxicillin allergy [14]. A similar recommendation may be proposed for amoxicillin + clavulanic acid. In contrast, the majority of the forms of paracetamol do not contained aspartame (*n* = 160/181, 88%). The simpler solution will be to propose these forms instead of those containing aspartame. Finally, we have to remember that the use with caution of Phe containing drugs may be limited to PKU patients requiring a strict dietary restriction of Phe whereas patients with relaxed diet can consume such medications without restriction.

In this analysis, several limitations can be noted, the first one is due to the calculating method used to determine the daily Phe-intake of each drug. This leads to a probable overestimation of the results since we considered that all the consumed aspartame will be converted in Phe. The second limitation was the different categories of daily Phe-intake (low, medium and high) that have been arbitrarily determined. However, the cut-off values were based on literature. The French Phe-exchange system for calculating Phe-intake is arbitrarily defined by 1 part of Phe = 20 mg of Phe [4]. The “low Phe intake” was defined by a daily Phe intake < 10 mg/d (0.5 part/d), which was considered as negligible amounts, equivalent to 5% or lower of the minimum daily Phe intake (the more severe dietary restriction is about 200 mg/d) [4]. From there, we proposed to define medium Phe-intake between 10 and 40 mg/d (0.5 to 2 parts/d), and high Phe intake as > 40 mg/d (> 2 parts/d.) In the recent PKU dietary handbook accompanying European PKU guidelines, an additional dose of 25–50 mg/d of Phe (approx. 0.5–1 g of natural protein) is proposed to significantly increasing Phe-intake to establish maximum Phe-tolerance [3]. In contrast, van Rijn and colleagues suggest that patients with well-controlled PKU can incidentally and punctually tolerate to double their normal daily Phe intake (from 360 to 2000 mg/d) without significant variation of their metabolic control [10]. Finally, another limitation is that this work has been designed based on the French list of medications established in 2020 and that this list is supposed to vary between countries and with years.

## Conclusion

To summarize, we demonstrated in this study that the number of drugs containing significant amounts of Phe or its precursor aspartame remained very limited. Among the aspartame containing drugs, only approximately half had a significant (medium or high) Phe intake whereas the other moiety brought negligible amounts of Phe. Furthermore, these medications with a high Phe intake were limited to few pharmaceutical classes (mainly antiinfectives agents, analgesics and drugs targeting central nervous system), and within these classes the drugs were limited to a small number of molecules, including principally amoxicillin, amoxicillin + clavulanic acid and paracetamol/ acetaminophen. In situations requiring the use of these molecules, we propose as an alternative, the use of an aspartame-free form of these molecules or a form with a low Phe intake. If it is not possible, we propose as second-line the use of another antibiotics or analgesics. Finally, we have to remember the benefit-risk balance to use medications containing significant Phe intake in PKU patients. Indeed, it may be better to use a Phe containing medication in the absence of an aspartame-free form of this drug rather than to leave a person with PKU without treatment. Additionally, note that in ageing adult patients with PKU, the presence of comorbidities may be frequent, and late-treated patients may be on anti-epilepsy drugs, both circumstances requiring chronic drug use. In such situations, all measures must be taken to optimise metabolic control, even in patients who have established brain damage, and significant aspartame-containing drugs may be avoided for a chronic use.

## Supplementary Information

**Additional file 1.** List of medications with and without aspartame or Phe. The list of drugs containing aspartame and/or Phe was established by using the French database “Theriaque”. The daily Phe intake of each drug was calculated using the drug composition, its conventional posology and the stoichiometric equation for aspartame metabolism. Results are presented according to age and weightand were distributed into 3 categories: “low Phe intake”, “medium Phe intake”and “high Phe intake”. Sheet 1 presented the list of all drugs with aspartame or Phe. Sheet 2, 3 and 4 presented the lists of the various forms of the 3 more frequent molecules containing aspartame, with for each drug the recommended forms according to weight or age.

## Data Availability

The list of drugs marketed in France containing aspartame and/or Phe was established by using the French database “Theriaque” (https://www.theriaque.org), accessed the 2021, April 21st, using the keywords “aspartame”, “phenylalanine” and “excipient”. The complete list is given in Additional file 1. The datasets used and analyzed during the current study are available from the corresponding author on reasonable request.

## Abbreviations

Phe: Phenylalanine
PKU: Phenylketonuria
NSAIDs: Non-steroidal anti-inflammatory drugs
